# Quality of life, cognitive and behavioural impairment in people with motor neuron disease: a systematic review

**DOI:** 10.1007/s11136-024-03611-5

**Published:** 2024-02-12

**Authors:** Ratko Radakovic, Chelsea Radakovic, Sharon Abrahams, Zachary Simmons, Amy Carroll

**Affiliations:** 1 Euan MacDonald Centre for Motor Neuron Disease Research, University of Edinburgh, Edinburgh, UK; 2 Alzheimer Scotland Dementia Research Centre, University of Edinburgh, Edinburgh, UK; 3 Cambridgeshire and Peterborough NHS Foundation Trust, Cambridge, UK; 4 Royal Papworth Hospital NHS Foundation Trust, Cambridge, UK; 5 Human Cognitive Neuroscience-Psychology, School of Philosophy, Psychology and Language Science, University of Edinburgh, Edinburgh, UK; 6 Department of Neurology, Pennsylvania State University, Hershey, PA USA; 7 Norfolk and Norwich University Hospital, Norwich, UK; 8 Department of Clinical Psychology and Psychological Therapies, Norwich Medical School, University of East Anglia, Norwich, NR4 7TJ UK

**Keywords:** Motor neuron disease, Behavioral symptoms, Neurobehavioral manifestations, Cognition, Cognition disorders, Quality of life, Systematic review

## Abstract

**Purpose:**

Motor neuron disease (MND) is a neurodegenerative disease, progressively impacting function and self-perceived quality of life (QoL). Up to 50% of people with MND can present with cognitive and behavioural impairment, with an associated increase in caregiver burden or strain. However, there has been no systematic exploration of the relationship between QoL and cognitive or behavioural impairment in MND. The aim was to determine if there is a relationship between QoL and cognitive/behavioural impairment in MND, while also supplementarily looking to determine the types of cognitive/behavioural and QoL measures utilised in these studies.

**Methods:**

A systematic search was performed across multiple databases (PsychINFO, Embase, Medline, AMED) for research published up to the date of February 22, 2023. Studies utilising quantitative methods of measuring QoL, cognitive/behavioural functioning/impairment were included. Findings examining relationships between QoL-cognitive/behavioural impairment were extracted and synthesised.

**Results:**

A total of 488 studies were identified, with 14 studies included in the systematic review. All 14 studies were observational (11 cross-sectional, 3 longitudinal). 13 studies utilised MND non-specific measures, particularly in relation to QoL and cognitive impairment. Of 8 studies measuring behavioural impairment 62.5% (*N* = 5) found either a lower QoL difference or association. Only 33.3% (*N* = 4) of 12 studies measuring cognitive impairment found a lower QoL difference or association.

**Conclusions:**

This systematic review shows that behavioural impairment may have an impact on QoL in MND. There is variability in types of assessments used to measure QoL and also cognitive/behavioural impairment, most of which are disease-non-specific. Recommendations for future research are to use comprehensive disease-specific, multidomain measures to further elucidate the QoL-cognitive/behavioural impairment relationship.

**Supplementary Information:**

The online version contains supplementary material available at 10.1007/s11136-024-03611-5.

## Introduction

Motor neuron disease (MND) [1] is an umbrella term that subsumes several different neurodegenerative conditions, such as primary lateral sclerosis (PLS), progressive bulbar palsy (PBP), progressive muscular atrophy (PMA), with the most prevalent of those being amyotrophic lateral sclerosis (ALS) [2, 3]. ALS and other forms of MND are progressive degenerative conditions that affects the central nervous system, impacting individuals’ physical functioning, characterised by muscle wasting of the upper and lower limbs, loss of functional abilities, including speech and movement, respiratory problems, loss of communicative abilities and autonomy [4]. The life expectancy from diagnosis is between two to five years.

Up to 15% of people with MND (pwMND) can develop frontotemporal dementia (FTD), often typified by progressive cognitive and behavioural impairment, and a lower insight/awareness for these symptoms, in addition to physical deterioration [5–7]. As MND and FTD exist on a disease spectrum [8–10], individuals without dementia can develop milder cognitive and behavioural impairment, occurring in up to 50% of pwMND throughout disease stages [11–13]. The most common cognitive impairments in MND are in executive functioning (particularly verbal fluency), language function and social cognition [14, 15]. These are most often assessed through neuropsychological batteries but commonly via cognitive screens specific to MND e.g. Edinburgh Cognitive and Behavioural ALS Screen (ECAS) cognitive screen, ALS Cognitive Behavioral Screen (ALS-CBS) cognitive subscale [16]. Previous research has shown that cognitive impairment, particularly executive dysfunction, can have a negative impact on functional decline, survival for pwMND [17, 18], and is associated with increased caregiver burden or strain [19].

The most common behavioural impairments are apathy (as a lack of motivation) and disinhibition [20, 21], which have been shown to occur across disease stages [22]. These are assessed through questionnaires, scales or semi structured behavioural interviews that are commonly specific to MND e.g. Beaumont Behavioural Inventory (BBI), ECAS behavioural interview. Previous research has shown that caregiver burden or strain is associated with behavioural impairments in MND, which can be further compounded by physical deterioration [23]. Behavioural impairment has also been shown to have a negative impact on disease progression [24] and ultimately on survival of pwMND [25, 26], particularly apathy [27].

As a progressive neurodegenerative disease, motor neuron disease can impact both the wellbeing or quality of life (QoL) of pwMND and also their caregivers or family members [28–30]. Caregiver burden or strain has been shown to associate with declining physical functioning of the pwMND, with a further emphasis on the caregivers mental health, particularly depression [31, 32]. Parallel to this, QoL for pwMND is also effected, due to associated loss of functioning as the condition progresses [29, 33, 34]. In terms of physical aspects of QoL relating to loss function, respiratory difficulties and communication, there are interventions available for management [35, 36]. Furthermore, psychological- or mental health-related QoL have been shown to be impacted in MND [30, 37], particularly in relating to hopelessness and social withdrawal [38, 39]. While there has been increasing utility of disease-specific, multidomain QoL instruments for MND [40], many studies still utilise generic, disease-non-specific QoL measures [41].

As such, while there has been research separately investigating cognitive or behavioural impairment and QoL in MND, there have been no systematic reviews exploring relationships between these factors. Furthermore, while use of QoL, cognitive and behavioural measures [16, 41] have been explored in MND, it is unclear what QoL measures (generic, disease-specific) have been used in the context of cognitive and/or behavioural functioning.

### Review aims

The systematic review primarily aimed to explore if there was an association between QoL and cognitive or behavioural impairment in MND. Further secondary aims were to determine what types of measures are used to explore QoL and cognitive or behavioural functioning in these studies.

### Methodology

The Preferred Reporting Items for Systematic reviews and Meta-Analyses (PRISMA) [42, 43] were followed in completion of this systematic review. The systematic review protocol was registered with the PROSPERO registry (https://www.crd.york.ac.uk/prospero/display_record.php?ID=CRD42022295512).

### Information sources

The following databases were searched systematically (periods of searches and data retrieval are specified in brackets next to each database): PsychINFO (1806 to 22nd February 2022), MEDLINE (1946 to 22nd February 2022), Embase (1947 to 22nd February 2022) and AMED (1985 to 22nd February 2022) via OVID. The searches were updated on 22nd February 2023 using the same databases. Backwards citation tracking was additionally applied to all full text articles that were reviewed.

### Search strategy and eligibility criteria

The searches included free text keyword terms (inclusive of spelling variations) and medical subject heading (MeSH) specific to each database. Search terms were linked by Boolean operators (i.e. AND, OR). The search terms were disease/condition of MND AND cognitive terms OR behaviour terms AND QoL terms. Exact search terms, free text keyword and MeSH search terms that were used, mapped to each database, can be found in Online Resource 1.

Eligibility criteria for inclusion of studies in the systematic review were primary and secondary quantitative research studies, with no restriction on specific quantitative study designs or on language, including individuals with an MND diagnosis, of 18 years or older. Studies required a quantitative measure of QoL or wellbeing, quantitative measure of cognitive and/or behavioural functioning. Exclusion criteria were qualitative studies (with no quantitative element), study population where individuals did not have an MND diagnosis, of 17 years old or younger. Editorials, conference abstracts, book chapters, other systematic reviews or meta-analyses were excluded.

### Study selection

Following PRISMA guidelines, Stage 1 involved two reviewers screening titles and abstracts independently based on the inclusion/exclusion eligibility criteria. Articles classified as relevant by both reviewers were included in the next stage of the review and articles deemed not relevant by both reviewers were excluded. Articles where two reviewers disagreed or that were classified ambiguous, progressed to Stage 2 of the review.

In Stage 2, the two reviewers examined full articles independently, applying the inclusion/exclusion eligibility criteria. Articles classified as relevant by both reviewers were included in the review and articles deemed not relevant by both reviewers were excluded. Articles where two reviewers disagreed on inclusion/exclusion or that were classified ambiguous were forwarded to a third reviewer for adjudication.

For the final included articles, all studies were examined for risk of bias/quality and all relevant data were extracted for synthesis. See below the Risk of bias (quality) assessment and Analysis sections for details of relevant quality scoring and data extracted methodology.

### Risk of bias (quality) assessment

Quality of individual studies was assessed using the National Heart, Lung, and Blood Institute (NHLBI) Study Quality Assessment Tools [44], covering a broad range of study designs and is a well-established, frequently updated tool in clinical literature [45]. These tools were used to determine the quality of various quantitative studies. Each item can be answered "Yes", "No", "Cannot Determine/Not Reported" and “Not Applicable”. If "No" is selected, the reviewers consider potential bias for this item. If "cannot determine" or "not reported" is selected, these may represent potential flaws in the study design, reporting and/or implementation. If “Not Applicable” is selected, this was accounted for in overall judgement of the quality/risk of bias. The quality review was completed by two reviewers independently, with studies classified as “good”, “fair” or “poor” based on consensus. The tool was adapted to assess cognitive and behavioural impairment assessment separately, with items 6 to 10 relating to exposure of interest (cognitive and behavioural impairment) being additionally subdivided.

### Analysis

Narrative synthesis of quantitative data from included studies was conducted to explore if there are any patterns of associations or differences in QoL in terms of cognitive and/or behavioural impairment.

Author (year), country, study design, sample size, age, sex, measures (QoL, cognitive and/or behavioural impairment) and results were extracted from included studies. In terms of results, examples of quantitative data that were synthesised were correlations, regressions and group differences. Specifically, if available, correlations (Pearson’s r, Spearman’s rho or other relevant metrics, including *p* values), regression results (beta coefficients or other relevant metrics, including *p* values) or group differences (t, F or other relevant metrics, including *p* values) for cognitive or behavioural measures relative QoL measures were extracted and synthesised. Furthermore, the overall robustness of the data synthesis was also analysed and considered in relation to quality of included studies (i.e. risk of bias).

## Results

### Search results

**Fig. 1:**
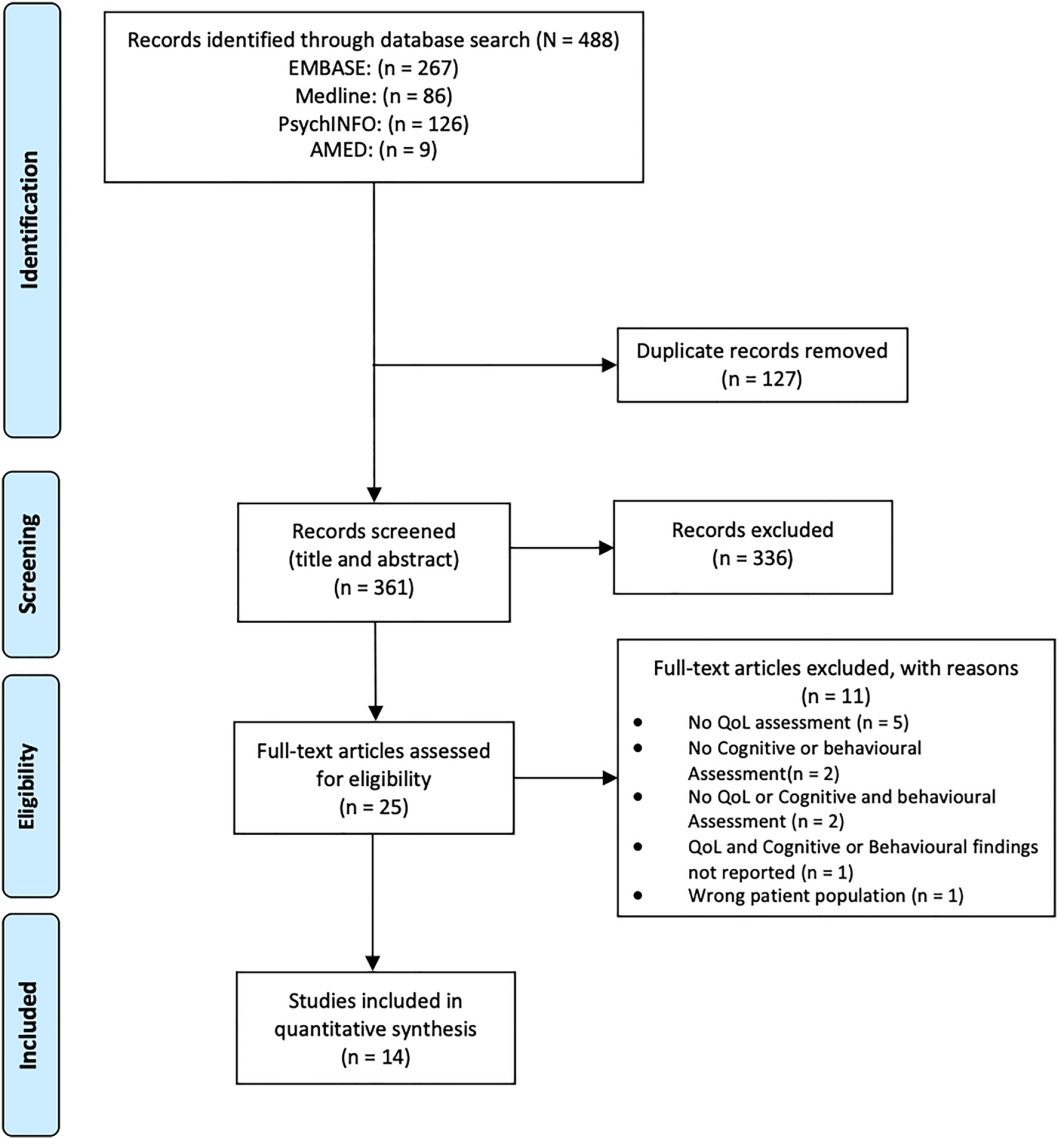
PRISMA Flowchart

Figure. 1 shows the PRISMA screening process [42, 43]. Following duplicate removal (*N* = 127), 488 potentially relevant articles were identified, with full text screening being performed on 25 articles. A total of 14 studies met eligibility criteria and were included in this review [46–59].

Table 1 presents a summary of the included studies in the systematic review. All 14 included studies were observational, with 78.6% (*N* = 11) being cross-sectional and 21.4% (*N* = 3) being longitudinal. The majority of studies recruited people with a diagnosis of ALS (64.3%, *N* = 9), while other studies recruited people with unspecified MND diagnoses or other forms of MND (e.g. primary lateral sclerosis, flail arm syndrome, those with co-occurring FTD). The total sample size from included studies was 1648 MND patients (range of 22 to 503), with a median sample size of 65 (Interquartile Range = 83.75). For the 12 studies reporting male–female ratio, 62.2% (*N* = 919) of MND patients were male.

**Table 1:** Summary of all included studies (*N* = 14)

*Author (year)*	*Country*	*Study Design*	*Diagnosis*	*N*	*Age (mean, SD)*	*Gender M/F*	*QoL measure(s)*	*CI Measure(s)*	*BI Measure(s)*	*Worse QoL-CI (Yes/No/NA)*	*Worse QoL-Bi (Yes/No/NA)*	*Quality Rating score*
Bock et al. (2016) [46]	USA	Cross-Sectional	ALS	86	63.8 (10.1)	50/36	**MQoL-Single Item Scale**	**ALS CBS Cognitive Subscale**	**ALS CBS Behavior Subscale**	No	No	Fair
Bock et al. (2017) [47]†	USA	Prospective Longitudinal	ALS	49	64.8 (11.1)	28/21	**MQoL-Single Item Scale**	**ALS CBS Cognitive Subscale**	**ALS CBS Behavior Subscale**	No	Yes	Fair
Caga et al. (2018) [48]	Australia	Cross-Sectional	ALS ALS-FTD	60	62.9 (1.3)	NR	**PWIA**	M-ACE	**AES-Self rated version** MiND-B	NA	Yes	Fair
Chio et al. (2010) [49]	Italy	Cross-Sectional	ALS	70	61.9 (10.0)	37/33	**MQoL Questionnaire**	MMSE	**FrSBe**	NA	No	Fair
Galvin et al. (2020) [50]	Ireland	Prospective Longitudinal	ALS	28	61.8 (8.8)	19/9	**SEIQoL-DW**	**ECAS Cognition**	**BBI**	No	No	Fair
Garcia-Willingham et al. (2018) [51] ††	USA	Cross-Sectional	ALS PLS	37	59.97 (11.37)	20/17	**MQoL Questionnaire-Psychological symptoms subscale**	**WCST**	**BRIEF-A-both Informant and self report versions**	No	Yes	Fair
Goldstein et al. (2002) [52]	UK	Cross-Sectional	MND	31	65.00 (11.85)	19/12	**SEIQoL-DW** SIP	**SIML**	None	Yes	NA	Poor
Gordon et al. (2010) [53]	USA	Longitudinal	ALS	50	56.0 (12.4)	25/25	**Q-LES-Q**	**Deficit score/impairment category (normal, mild, moderate) calculated from neuropsychological assessment battery (WCST, SCWT, RVLT, BVRT, DSF, DSB, BNT, COWA including PF, SF, WTAR)**	None	No	NA	Fair
McCabe et al. (2010) [54]	Australia	Cross-Sectional	MND	120	62.06	NR	**WHOQOL-BREF**	**SS- concentration difficulties**	None	Yes	NA	Poor
Prell et al. (2020) [55]	Germany	Cross-Sectional	ALS	125	64 (16)**	73/52	**ALSAQ-40**	**ECAS Cognition**	None	No	NA	Fair
Rabkin et al. (2016) [56]	USA	Cross-Sectional	ALS	253	60.4 (9.8)	149/104	**Q-LES-Q** **Custom designed QoL VAS**	**ALS CBS Cognitive Subscale** MMSE	**ALS CBS Behavior Subscale** ALS-FBI	No	Yes	Fair
Schrempf et al. (2021) [57]	Germany	Cross-Sectional	ALS PBP PLS PMA FAS	214	60.1 (12.5)	130/84	**SEIQoL-DW** **ACSA**	**ECAS Cognitive**	**ECAS Behavioural**	No	Yes	Fair
Trojsi et al. (2016) [58]	Italy	Cross-Sectional	ALS	22	59.19 (9.63)	13/9	**SF-36**	**EAT** ACE-R RCPM TT MPT SEF ATT ET	FrSBe	Yes	NA	Fair
Wei et al. (2021) [59]	China	Cross-Sectional	ALS	503	54.7 (11.4)	347/156	**EQ-5D-5L**	**ACE-R** **FAB**	None	Yes	NA	Fair

Quality of life, behaviour and/or cognitive variable relationships examined in each study are highlighted in bold in the Table

*QoL* Quality of Life, *N* Number, *M* Male, *F* Female, *SE* Standard Deviation, *CI* Cognitive Impairment, *BI* Behavioural Impairment, *USA* United States of America, *UK* United Kingdom, *ALS* Amyotrophic lateral sclerosis, *PLS* Primary Lateral Sclerosis, *PBP* Progressive Bulbar Palsy, *PMA* Primary Muscle Atrophy, *FAS* Flail Arm Syndrome, *WCST* Wisconsin Card Sorting Test, *SCWT* Stroop Color and Word Test, Memory, *RVLT* The Rey Verbal Learning Test, *BVRT* Benton Visual Recognition Test, *DSB* Digit Span Backward (working memory), *BNT* Boston Naming Test, *COWA* Controlled Oral Word Association COWA, *PF* Phonemic FAS fluency, *SF* semantic fluency (animal naming), *DSF* Digit Span Forward, *WTA* The Wechsler Test of Adult Reading, *ACE-R* Addenbrooke’s Cognitive Examination Revised, *FAB* Frontal Assessment Battery, *RCPM* Raven’s colored progressive matrices, *TT* Token Test, *MPT* Memory Prose Test, *SEF* Stroop Executive Factor, *EAT* Emotional Attribution Task, *ATT* Avoidance Test of Theory of Mind, *ET* Eyes Test, *FrSBe* Frontal Systems Behavior Scale, *ECAS* Edinburgh Cognitive and Behavioural ALS Screen, *ALS CBS* ALS Cognitive Behavoral Screen, *ALS-FBI* ALS Frontal Behavior Inventory, *MMSE* Mini Mental State Exam, *M-ACE* Mini-Addenbrooke’s Cognitive Examination, *MiND-B* Motor Neuron Disease Behavioral Scale, *AES* Apathy Evaluation Scale, *BBI* Beaumont Behavioural Inventory, *BRIEF-A* Behavior Rating Inventory of Executive Functions adult version, *SS* Symptom Scale, *SIML* Short Inventory of Minor Lapses, MQoL McGill QoL, *PWIA* Personal Wellbeing Index-Adult, *SEIQoL-DW* Schedule for the Evaluation of the Individual Quality of Life-Direct Weighting, *SIP* Sickness Impact Profile, *ACSA* Anamnestic Comparative Self-Assessment, *VAS* Visual Analogue Scales, *Q-LES-Q* Endicott's Quality of Life Enjoyment and Satisfaction Questionnaire, *EQ-5D-5L* Five-level EuroQol-5 dimensions, *SF-36* Short Form-36, *ALS-AQ40* ALS Assessment Questionnaire, *WHOQOL-Bref* World Health Organization Quality of Life questionnaire

^*^Pooled

^**^Median (IQR)

^†^Baseline characteristic previously reported in [46]

^††^Supplementary data provided by author

### Summary of risk of bias/quality assessment

Overall, the studies included in this review were of a “Fair” quality. The aims, eligibility criteria and validity of behavioural measures were clear across studies, for both cross-sectional and longitudinal studies. Study population showed heterogeneity through inclusion of other forms of MND, as well as ALS. Further issues and difficulties were related to eligibility rates (either due to being less than 50% or not reported) and sample size justification, inclusive of inconsistent statistical reporting. Furthermore, validity of cognitive measures were found to affect the study quality due to non-disease specificity, with an overall variability of classification and measurement for both behavioural and cognitive impairment. For QoL outcome measures, non-disease-specific measurement was observed as an issue. Complete quality ratings for included studies can be found in Online Resource 2.

### Behavioural impairment and QoL

A total of 8 studies explored behavioural impairment in relation to QoL (Online Resource 3 shows full statistical findings). Of those 62.5% (*N* = 5) found either a QoL difference or association relative to behavioural srimpairment. Rabkin et al. [56] found that those grouped as having behavioural impairment had significant lower QoL than those with no behavioural impairment (using disease-specific measures). This same study further observed that pwMND with behavioural impairment had lower QoL across a variety of subdomains (assessed by custom designed single-item QoL-Visual Analogue Scale), specifically worse mental health concerns (anxiety/depression), increased weariness, more suffering, lower religion/spirituality and more hopelessness.

A study using two generic, disease-non-specific QoL measures found conflicting results where one measure, the Anamnestic Comparative Self-Assessment (ACSA), showed those with behavioural impairment had significantly lower QoL than those without behavioural impairment, but the other measure, the Schedule for the Evaluation of Individual Quality of Life-Direct Weighting (SEIQoL-DW) did not show this difference [57].

Further, a longitudinal study by Bock et al. [47] using disease-specific measures showed that those with behavioural impairment showed a significant decline in QoL across two time points (6.8 months apart), even when controlling for age, sex, region of onset, functional disability, respiratory difficulties and depressive symptoms. Further exploration showed that worsening QoL related to worsening apathy and irritability [47]. However, a previous analysis of just the baseline visit from the above mentioned longitudinal study found no significant association between QoL and behavioural impairment at a cross-sectional level [46].

A further study showed that those with apathy (assessed using a disease, non-specific measure) had significantly lower overall QoL, lower QoL in achieving in life and community connectedness subdomains [48]. The same study found when controlling for depression, emotional apathy was a negative predictor of achieving in life and community connectedness. Additionally, another study showed an association indicative of lower psychological QoL and lower self-rated behavioural regulation using a disease-non-specific measure [51].

Finally, one study utilising disease-specific measure the BBI [50], as well as one study using a disease-non-specific measure the FrSBe [49] found no behavioural impairment association or difference relative to QoL.

### Cognitive impairment and QoL

Of 12 studies, only 33.3% (N = 4) found relationships between cognitive impairment and QoL (see Online Resource 3 for full statistical findings). Wei et al. [59] found a correlation between a generic, disease-non-specific cognitive screening measure, the Addenbrooke’s Cognitive Examination Revised (ACE-R), of cognitive functioning and disease-non-specific QoL measure (EQ-5D-5L), indicative of better QoL associating with better cognitive functioning. However, this was not observed when grouping individuals based on higher and lower ACE-R score. Additionally, this same study using the Frontal Assessment Battery (FAB; a disease-non-specific executive functioning measure) found that pwMND with lower executive functioning had lower QoL on the EQ-5D-5L.

A different study found a positive correlation between a specific cognitive task exploring theory of mind (Emotion Attribution Task) and mental health-related QoL (disease-non-specifical measure), indicative of better QoL associated with better theory of mind [58]. Two studies utilised generic, self-rated, disease-non-specific cognitive symptom measures, the Short Inventory of Minor Lapses (SIML) and Symptoms Scale–Cognitive, found significant correlations, indicating more cognitive problems associating with lower generic, disease-non-specific QoL [52, 54].

The remaining 66.7% (*N* = 8) studies found no relationship between cognitive impairment and QoL. Notably, these studies all used disease-specific instruments (i.e. ECAS, ALS-CBS, neuropsychological test batteries) [46, 47, 50, 51, 53, 55–57]. The only study that utilised a disease-specific QoL instrument, the ALS Assessment questionnaire-40 (ALSAQ-40), found that while cognitive subdomain impairments (specifically visuospatial, executive and fluency) were significant negative predictors of emotional well-being-related QoL, this was no longer significant when controlling for depression, hopelessness and pain [55].

### Behavioural and cognitive measurement

Of the 14 studies, 42.9% (*N* = 6) explored both cognitive and behavioural impairment [46, 47, 50, 51, 56, 57]. Further, 42.9% (*N* = 6) of studies looked at cognitive impairment only [52–55, 58, 59] and 14.2% (*N *= 2) looked at behavioural impairment only [48, 49].

Of 8 studies utilising behavioural assessments, 62.5% (*N *= 5) used disease-specific behavioural screening instruments such as the ECAS behavioural interview [57], ALS-CBS behavioural subscale [46, 47, 56] and the BBI [50]. The remaining three studies used disease-non-specific instruments such as the Frontal Systems Behavior Scale (FrSBe) [49], the Apathy Evaluation Scale (AES) [48] and the Behavior Rating Inventory of Executive Functions adult version (BRIEF-A) [51]. There were two studies that used two measures of behavioural impairment. Caga et al. [48], the AES and Motor Neuron Disease Behavioral Scale (MiND-b), but did not report any results relating to the latter measure and QoL. Similarly Rabkin et al. [56] utilised the ALS Frontal Behavior Inventory (ALS-FBI), as well as the ALS-CBS behavior subscale, but once again did not report any results relating to QoL. Finally, one study utilised the FrSBe but did not report results relating to QoL [58].

Of the 12 studies utilising cognitive assessments, 50.0% (*N* = 6) used disease-specific cognitive screening instruments, with three studies using the ECAS [50, 55, 57] and three studies using the ALS-CBS cognitive subscale [46, 47, 56]. A further 25.0% (*N* = 3) studies used neuropsychological batteries or specific cognitive tests (executive functioning or theory of mind) appropriate for MND [51, 53, 58]. One study used two disease-non-specific cognitive measures, the ACE-R and the FAB [59].

### QoL measurement

While all QoL measures used in included studies were self-rated, 92.9% (*N* = 13) studies employed generic, disease-non-specific QoL measures [46–54, 56–59]. The two most common generic, disease-non-specific QoL measures utilised were the McGill QoL (MQoL) full questionnaire or single-item measure (*N* = 4), the SEIQoL-DW instrument (*N* = 3). Only one study used a disease-specific QoL measure which was the ALSAQ-40 [55].

Notably, while 78.6% (*N* = 11) of studies only used one measure of QoL, in addition to using one of the measures listed above, three studies used an additional (second) QoL measure [52, 56, 57]. These additional measures were the ACSA, multiple custom designed single-item QoL-Visual Analogue Scale assessing different subdomains, and the Sickness Impact Profile (SIP), all of which were disease-non-specific. 50% (*N* = 7) of studies used methods or measures that could examine different QoL domains [48, 49, 54–56, 58, 59].

## Discussion

This systematic review shows that there may be a relationship between self-perceived QoL and behavioural impairment, in the context of variable QoL, cognitive and behavioural measurements that were used. About a third of studies that measured behaviour found that there was an associated effect between this impairment and lower QoL.

These findings build on previous research suggesting that behavioural impairment is associate with strain, burden and distress for caregivers or family members [31]. The impact of behavioural impairment may extend towards the pwMND themselves, through overall behavioural impairment negatively affecting QoL. Further to this, there is indication that certain behavioural domains such as apathy, irritability and behavioural-regulation (akin to disinhibition) may relate to lower QoL. Apathy as the most common behavioural impairment in MND [20] may result in withdrawal from everyday life, family gatherings and social events. Caga et al. [48] found that pwMND with apathy had lower QoL relating to community connectiveness and satisfaction in life, particularly relative to emotional elements of apathy. Emotional apathy (as emotional neutrality/indifference towards self, others and surroundings), has been shown to be more characteristic and prevalent in FTD [60–62]. Emotional apathy may have overlap with diminished sympathy and empathy features that are observed in FTD and milder behavioural impairments in MND. These types of behavioural impairments may represent observable changes in the pwMND through how they interact with people in the environment and how those people reciprocally interact with them. This could result in a negative dynamic between the pwMND and the others around them, reverberating as a negative impact on QoL. As such there might be a complex interplay between self-perceived QoL, interaction and caregiver burden or strain in relation to behavioural impairment, which should be an avenue for future research. Notably studies that did detect QoL-behavioural impairment links in this systematic review predominantly utilised disease-specific measures, for example the ECAS and the ALS-CBS, whereas those that did not used disease-non-specific measures. More recently systematic reviews of measurement have propagated the consistent use of disease-specific measures of cognitive functioning and behaviour in both exploratory research but also in clinical trial research [16, 63, 64]. This in combination with our findings emphasises further the importance of disease-specific measurement of behavioural impairment in MND.

For cognitive impairment, the studies in this systematic review overall that did not find a robust QoL association, utilised either comprehensive cognitive screens or neuropsychological assessment/tasks that were disease-specific. Associations between cognitive impairment and lower QoL were only found in one third of studies, most of which were to do with overall cognitive functioning and were assessed by disease-non-specific measures such as self-rated questionnaire measures or generic cognitive screens (e.g. SIML, ACE-R, FAB). While self-rated cognitive questionnaires give insight to cognitive functioning, they may also capture subjective perception of cognitive impairment and worry expressed by the individuals completing the measures. This may therefore induce bias of over-judgement relating to cognitive difficulties and confound any cognitive functioning-QoL relationships. The current criteria for detecting cognitive impairment recommends objective, quantifiable cognitive screening or neuropsychological assessments/tasks [9, 10]. The findings seem to suggest that objectively assessed frontal executive or theory of mind-related impairments associates with worse QoL [58, 59]. These types of cognitive impairments have been observed to be common in MND [65]. Further, these cognitive domains are important for higher order processing and social interaction or understanding, as such these may impact people’s confidence in performing tasks independently and interacting with the outside world, which may result in a knock-on effect on QoL.

In terms of QoL assessment, the measures used across studies study were predominantly generic and disease-non-specific. While some of the most common measures used were the MQoL and SEIQoL-DW, the total score of the latter may not be representative of the disease-specific experience of pwMND and have variable intercorrelations with other QoL measures in MND [66, 67]. These types of items may not quantifiably capture what is important for pwMND in the context of their condition. Disease-specific measures such as the ALSAQ-40 or the ALS-Specific QoL (ALSsQoL) instrument explore domains that are relevant to MND, for example for communication, intimacy, eating, interaction with the environment and mental health. Moreover, in studies where QoL was explored multidimensionally, this yielded meaningful findings about the experiences of pwMND in relation to QoL associated with mental health and social connectedness. As such, this is supportive of disease-specific measures being used for assessment of QoL in MND.

In assessing included studies, there was apparent reporting and methodological limitations, either in relation to sample size, eligibility, study population or statistical reporting. While different types of MND share clinicopathological similarities, they can have varying rates of progressions and regions effected. As such this could variably impact self-perceived QoL dependent on the type of MND, and may have a complex interaction with cognitive or behavioural profiles. Of further note is that more objective measures of cognitive functioning could have been used. All these factors impact the quality of included studies, increase the chance of bias resulting in cautious interpretation of the findings of this systematic review.

### Recommendations

The core recommendation for future research centre around study population, more standardised reporting of methods and statistics, consistent design and identification of cognitive and behavioural impairment (both longitudinally and cross-sectionally) as well as more targeted application of MND-specific measures, across QoL, cognition and behaviour. In particular, future research should use disease specific, multidimensional cognitive and behavioural screen (i.e. ECAS) to determine impairments. Ideally, individuals should undergo assessment using a comprehensive neuropsychological battery exploring-specific cognitive domains and thorough behavioural as well as neuropsychiatric assessment to reliably determine impairments for pwMND. Notably few studies explicitly applied the criteria for classification of cognitive and behavioural impairment in MND [9, 10], which may be further confound relative to consistency of findings and would be an avenue for future research.

### Strengths and limitations

A strength of this systematic review is that it was able to extract and meaningfully interpret findings from a relatively heterogeneous collection of included studies that utilised variable measurements methods of cognitive or behavioural impairment and QoL. However, this systematic review does have its own limitations. Due to the different study designs (longitudinal, cross-sectional) in combination with the differential reporting of and types of statistical analysis used in the included studies, it was not possible to perform a more in-depth synthesis of results (i.e. meta-analysis). As such, as the field develops further, it might provide opportunity for more in-depth analysis of the relationship between QoL-cognitive and behavioural impairment in MND.

## Conclusions

A collation of previous research from this systematic review suggests that behavioural impairment may be associated with worse QoL for pwMND. Future research utilising disease-specific, multidimensional instruments is required to further elucidate the characteristics of this complex relationship. A further benefit in utility of disease-specific instruments, would allow for more standardised comparison of differential impacts of cognitive and behavioural domains relative to QoL for pwALS. Understanding this might help guide further support for pwMND experiencing these difficulties, their families and the systems around them that can help with these difficulties. Consistent identification of specific cognitive-behavioural links with QoL will also help lay a baseline for interventional research that can help with person-centred understanding and care.

### Supplementary Information

Below is the link to the electronic supplementary material.

Supplementary file1 (PDF 93 KB)

Supplementary file2 (PDF 104 KB)

Supplementary file3 (PDF 57 KB)
