# Supplementary material for: Quality of life, cognitive and behavioural impairment in people with motor neuron disease: a systematic review
Source: Qual Life Res. 2024 Feb 12;33(6):1469–80. doi: 10.1007/s11136-024-03611-5 (PMC11116232; doi:10.1007/s11136-024-03611-5)
Supplement: Supplementary file 1 — Supplementary file1 (PDF 93 KB) [file 11136_2024_3611_MOESM1_ESM.pdf]

Online Resource 2. Quality assessment results details

Study	Study Type	1	2	3	4	5	6a	6b	7a	7b	8a	8b	9a	9b	10a	10b	11	12	13	14
Bock et al 2016	OC	Yes	Yes	Yes	Yes	No	No	No	No	No	NA	NA	Yes	Yes	NA	NA	Yes	NA	NA	Yes
Bock et al 2017	LOC	Yes	Yes	Yes	Yes	No	NR	NR	CD	CD	Yes	Yes	Yes	Yes	Yes	Yes	Yes	NA	No	Yes
Caga et al 2018	OC	Yes	No	NR	Yes	No	NA	No	NA	No	NA	NA	NA	CD	NA	NA	CD	NA	NA	Yes
Chio et al 2010	OC	Yes	Yes	NR	Yes	No	NA	No	NA	No	NA	NA	NA	Yes	NA	NA	CD	NA	NA	CD
Galvin et al 2020	LOC	Yes	Yes	NR	Yes	No	NR	NR	CD	CD	Yes	Yes	Yes	Yes	Yes	Yes	CD	NA	No	No
Garcia-Willingham et al 2018	OC	Yes	No	NR	Yes	Yes	No	No	No	No	NA	NA	Yes	No	NA	NA	CD	NA	NA	No
Goldstein et al 2002	OC	Yes	CD	NR	CD	No	No	NA	No	NA	NA	NA	No	NA	NA	NA	CD	NA	NA	Yes
Gordon et al 2010	LOC	Yes	Yes	No	Yes	Yes	NR	NA	CD	NA	Yes	NA	CD	NA	Yes	Yes	CD	NA	Yes	Yes
McCabe et al 2010	OC	Yes	No	CD	CD	No	No	NA	No	NA	NA	NA	CD	NA	NA	NA	CD	NA	NA	CD
Prell et al 2020	OC	Yes	Yes	NR	Yes	No	No	NA	No	NA	NA	NA	Yes	NA	NA	NA	Yes	NA	NA	No
Rabkin et al 2016	OC	Yes	Yes	No	Yes	Yes	No	No	No	No	NA	NA	Yes	Yes	NA	NA	CD	NA	NA	Yes
Schrempf et al 2021	OC	Yes	No	CD	Yes	No	No	No	No	No	NA	NA	Yes	Yes	NA	Na	CD	NA	NA	Yes
Trojsi et al 2016	OC	Yes	Yes	CD	Yes	Yes	No	NA	No	NA	NA	NA	CD	NA	NA	NA	CD	NA	NA	CD
Wei et al 2021	OC	Yes	Yes	CD	Yes	No	No	NA	No	NA	NA	NA	No	NA	NA	NA	CD	NA	NA	CD

OC= Observational Cohort, LOC= Longitudinal Observational Cohort, CD= Cannot Determine, NA= Not Applicable, NR= Not Reported

Quality assessment questions = 1. Was the research question or objective in this paper clearly stated?; 2. Was the study population clearly specified and defined?; 3. Was the participation rate of eligible persons at least 50%?; 4. Were all the subjects selected or recruited from the same or similar populations (including the same time period)? Were inclusion and exclusion criteria for being in the study prespecified and applied uniformly to all participants?; 5. Was a sample size justification, power description, or variance and effect estimates provided?; 6a. For the analyses in this paper, were the exposure(s) of interest measured prior to the outcome(s) being measured? (exposure cognitive impairment); 6b. For the analyses in this paper, were the exposure(s) of interest measured prior to the outcome(s) being measured? (exposure behavioural impairment); 7a. Was the timeframe sufficient so that one could reasonably expect to see an association between exposure and outcome if it existed? (exposure cognitive impairment); 7b. Was the timeframe sufficient so that one could reasonably expect to see an association between exposure and outcome if it existed? (exposure behavioural impairment); 8a. For exposures that can vary in amount or level, did the study examine different levels of the exposure as related to the outcome (e.g., categories of exposure, or exposure measured as continuous variable)? (exposure cognitive impairment); 8b. For exposures that can vary in amount or level, did the study examine different levels of the exposure as related to the outcome (e.g., categories of exposure, or exposure measured as continuous variable)? (exposure behavioural impairment); 9a. Were the exposure measures (independent variables) clearly defined, valid, reliable, and implemented consistently across all study participants? (exposure cognitive impairment); 9b. Were the exposure measures (independent variables) clearly defined, valid, reliable, and implemented consistently across all study participants? (exposure behavioural impairment); 10a. Was the exposure(s) assessed more than once over time? (exposure cognitive impairment); 10b. Was the exposure(s) assessed more than once over time? (exposure behavioural impairment); 11. Were the outcome measures (dependent variables) clearly defined, valid, reliable, and implemented consistently across all study participants?; 12. Were the outcome assessors blinded to the exposure status of participants?; 13. Was loss to follow-up after baseline 20% or less?; 14. Were key potential confounding variables measured and adjusted statistically for their impact on the relationship between exposure(s) and outcome(s)?
