# Supplementary material for: Quality of life, cognitive and behavioural impairment in people with motor neuron disease: a systematic review
Source: Qual Life Res. 2024 Feb 12;33(6):1469–80. doi: 10.1007/s11136-024-03611-5 (PMC11116232; doi:10.1007/s11136-024-03611-5)
Supplement: Supplementary file 2 — Supplementary file2 (PDF 104 KB) [file 11136_2024_3611_MOESM2_ESM.pdf]

Online Resource 3. Systematic Review full statistical findings for included studies (N=14)

Author (year)	*Findings*
Bock et al 2016 [38]	No significant association between cognitive (adjusted beta= -0.05, CI= -0.27, 0.17) or behavioral (adjusted beta= 0.03, CI= -0.03, 0.09) subscores and QoL in univariate or multivariate regression analyses (adjusted for forced vital capacity, age, education, gender, region of onset, Geriatric Depression Screen score, and ALSFRS-R.)
Bock et al 2017 [39]†	No change in QoL in those with cognitive impairment.<br><br>Significant decline in QoL in those with behavioural impairment (t=-4.3, p = .003). Significant change in QoL was associated with behavioural subscore (adjusted beta= 0.12, p=.009), when controlling for age, sex, region of onset, ALSFRS-R, FVC and depressive symptoms. Apathy (beta= 2.0, p=.001) and irritability (beta= 1.3, p=.001) were associated with decreased QoL.
Caga et al 2018 [40]	Patients with apathy (behavioural impairment) had lower overall QoL (t(56) = 3.4, p = 0.001), achieving in life subscore (t(56) = 3.6, p = 0.001) and community-connected subscore (t(56) = 4.1, p = 0.0001). Additionally, when controlling for depression subfactors of emotional manifestations of apathy were significant predictors of achieving in life subscore (beta = −6.4, p = 0.033) and community-connected subscore (beta = −7.0, p = 0.018).
Chio et al 2010 [41]	Patient's MQoL and depression scores were not correlated to their neurobehavioral symptoms (data not shown).
Galvin et al 2020 [42]	For patients median SIS was not statistically different according to cognitive status: (U = 46, P = .321) time 1; (U = 62, p = .658) time 2; (U = 76; P = .797) time 3.<br><br>No difference in median SIS due to presence/ absence of impairment for … patients… Patients: (U = 70, P = .912) at time 1; (U = 70, p = .598) time 2; (U = 70; P = .517) time 3.
Garcia-Willingham et al 2018 [43]††	Self-rated BRIEF Behavioral Regulation Index T score significantly negatively correlated with MQOL (r(35)= -0.381, p < .022).<br><br>No significant correlations between MQOL and WCST (Perseverative errors T score r(35)= 0.247, p = .393; Total errors T score r(35)= 0.092, p = .593).
Goldstein et al 2002 [44]	SEIQoL-DW scores showed a significant negative correlation with SIML scores (r = -0.434, p = 0.015), indicating that higher levels of cognitive lapses were associated with lower QoL scores… even when age was partialled out, SIML and SEIQoL-DW scores still demonstrated a significant negative correlation (r = -0.447, p = 0.013).<br><br>SIP relationship with SIML not reported.
Gordon et al 2010 [45]	No difference on deficit score/impairment category (cognitive impairment) categories on Q-LES-Q (F(2, 47)=0.52, p = .60.
McCabe et al 2010 [46]	Cognitive symptoms significant predictors of QoL (zero order correlation = -0.59, p < .001, beta -0.39.)
Prell et al 2020 [47]	ECAS total score (or specific cognitive domains) were not a significant predictor of the ALSAQ-40 summary index, domains mobility, ADL, eating, communication, and emotional well-being (when controlling for depression, hopelessness and pain).
Rabkin et al 2016 [48]	No significant difference between those with no cognitive impairment, with minor cognitive impairment and moderate impairment on Q-LES-Q (F(2,236)=0.46, p= .629). and VAS-QoL.<br><br>Significant difference between those with no behavioural impairment and with behavioural impairment on Q-LES-Q (t(238)=19.65, p< .001, effect size = 0.61) and on the VAS-QoL. Specifically VAS-QoL feeling depressed (t(231)=13.47, p< .001, effect size = 0.49), suffering (t(231)=6.88, p= .009, effect size = 0.38), weary (t(231)=7.67, p= .006, effect size = 0.38), nervous/anxious (t(231)=8.53, p= .004, effect size = 0.43), hopeful (t(231)=4.96, p= .027, effect size = 0.29), importance of religion/spirituality (t(231)=7.34, p= .007, effect size = 0.32) and overall (t(231)=9.17, p< .003, effect size = 0.42).

Schrempf et al 2021 [49]	No difference between ALSci and those without cognitive impairment on SEIQoL-DW (U = -0.79; p = 0.43; r = 0.08) and ACSA (U = -1.59; p = 0.11; r = 0.17).<br><br>Significant difference between ALSbi and those without behavioural impairment on the ACSA (U = -2.04; p = 0.04; r = 0.16) and not the SEIQoL-DW (U = -1.30; p = 0.20; r = 0.25,).
Trojsi et al 2016 [50]	Significant positive correlation between SF-36 Mental health and EAT (r= 0.570, p= 0.033; uncorrected level of significance).
Wei et al 2021 [51]	Significant difference on higher (≥ 16) and lower (< 16) FAB groups on both the EQ-5D-5L health utility index (*p* < .017) and the VAS score (*p* < .0.26).<br><br>Significant correlation between ACE-R (cognitive functioning) and the EQ-5D-5L health utility index values (r= 0.120, p= 0.005) and the VAS score (r= 0.127, p= 0.003).

GREEN indicated significant behavioural impairment-QoL finding. **BLUE** indicates significant cognitive impairment-QoL finding. **RED** indicates no cognitive or behavioural impairment-QoL finding.

†Baseline characteristic previously reported in Bock et al 2016

††Supplementary data provided by author

QoL= Quality of Life; N= Number; M= Male; F= Female; SE= Standard Deviation; CI= confidence interval; ALS= Amyotrophic lateral sclerosis, PLS= Primary Lateral Sclerosis, PBP= Progressive Bulbar Palsy, PMA= Primary Muscle Atrophy, FAS= Flail Arm Syndrome, WCST= Wisconsin Card Sorting Test; SCWT= Stroop Color and Word Test; Memory: RVLT= The Rey Verbal Learning Test, BVRT= Benton Visual Recognition Test, DSB=Digit Span Backward (working memory); BNT= Boston Naming Test; COWA= Controlled Oral Word Association COWA; PF Phonemic FAS fluency; SF= semantic fluency (animal naming); DSF= Digit Span Forward; WTA= The Wechsler Test of Adult Reading; ACE-R= Addenbrooke's Cognitive Examination Revised; FAB= Frontal Assessment Battery; RCPM= Raven's colored progressive matrices; TT= Token Test; MPT= Memory Prose Test; SEF= Stroop Executive Factor; EAT= Emotional Attribution Task; ATT= Avoidance Test of Theory of Mind; ET= Eyes Test, FrSBe= Frontal Systems Behavior Scale; ECAS= Edinburgh Cognitive and Behavioural ALS Screen; ALS CBS= ALS Cognitive Behavoral Screen; ALS-FBI= ALS Frontal Behavior Inventory; MMSE= Mini Mental State Exam; M-ACE= Mini-Addenbrooke's Cognitive Examination; MiND-B= Motor Neuron Disease Behavioral Scale; AES= Apathy Evaluation Scale; BBI= Beaumont Behavioural Inventory; BRIEF-A= Behavior Rating Inventory of Executive Functions adult version; SS= Symptom Scale; SIML= Short Inventory of Minor Lapses; MQoL= McGill QoL; PWIA= Personal Wellbeing Index-Adult; SEIQoL-DW= Schedule for the Evaluation of the Individual Quality of Life-Direct Weighting; SIP= Sickness Impact Profile; ACSA= Anamnestic Comparative Self-Assessment; VAS= Visual Analogue Scales; Q-LES-Q= Endicott's Quality of Life Enjoyment and Satisfaction Questionnaire; EQ-5D-5L= Five-level EuroQol-5 dimensions; SF-36= Short Form-36; ALS-AQ40= ALS Assessment Questionnaire; WHOQOL-Bref= abbreviated World Health Organization Quality of Life questionnaire
