# Supplementary material for: Quality of life, cognitive and behavioural impairment in people with motor neuron disease: a systematic review
Source: Qual Life Res. 2024 Feb 12;33(6):1469–80. doi: 10.1007/s11136-024-03611-5 (PMC11116232; doi:10.1007/s11136-024-03611-5)
Supplement: Supplementary file 3 — Supplementary file3 (PDF 57 KB) [file 11136_2024_3611_MOESM3_ESM.pdf]

Online Resource 1. Systematic Review search strategy

	Disease/Condition Term	AND	Cognitive/behaviour Term	AND	Quality of life Term
**Generic terms (across databases)**	“amyotrophic lateral sclerosis”<br>“motor neuron* disease”<br>“Lou Gehrig*”		“cogniti*”<br>“behav*”		“quality of life”<br>“QoL”<br>“life quality”<br>“wellbeing"<br>“well being”<br>“well-being”<br>“life satisfaction”
**PsychINFO specific (MESH)**	Amyotrophic Lateral Sclerosis		Cognition<br>Cognitive Ability<br>Cognitive Impairment<br>Behavior<br>Behavior Problems<br>Behavior Change<br>Behavior Disorders		Quality of Life<br>Health Related Quality of Life<br>Well Being<br>Life Satisfaction
**Medline specific (MESH)**	Motor Neuron Disease<br>Amyotrophic Lateral Sclerosis		Cognition<br>Cognition Disorders<br>Cognitive Dysfunction<br>Problem Behavior<br>Behavior		Quality of Life<br>Personal Satisfaction
**EMBASE specific (MESH)**	amyotrophic lateral sclerosis<br>motor neuron disease		cognition<br>cognitive defect<br>behavior<br>behavior change<br>behavior disorder		quality of life<br>wellbeing<br>life satisfaction
**AMED specific (MESH)**	Motor neuron disease<br>Amyotrophic lateral sclerosis		Cognition disorders<br>Cognition<br>Behavioral disorders		Quality of life
